# BTK inhibition limits B-cell–T-cell interaction through modulation of B-cell metabolism: implications for multiple sclerosis therapy

**DOI:** 10.1007/s00401-022-02411-w

**Published:** 2022-03-18

**Authors:** Rui Li, Hao Tang, Jeremy C. Burns, Brian T. Hopkins, Carole Le Coz, Bo Zhang, Isabella Peixoto de Barcelos, Neil Romberg, Amy C. Goldstein, Brenda L. Banwell, Eline T. Luning Prak, Michael Mingueneau, Amit Bar-Or

**Affiliations:** 1 The Center for Neuroinflammation and Experimental Therapeutics and the Department of Neurology, Perelman School of Medicine, University of Pennsylvania, Philadelphia, PA 19104 USA; 2 MS Research Unit, Biogen, Cambridge, MA 02142 USA; 3 Medicinal Chemistry, Biogen, Cambridge, MA 02142 USA; 4 Division of Immunology and Allergy, The Children’s Hospital of Philadelphia, University of Pennsylvania, Philadelphia, PA 19104 USA; 5 Department of Cardiology, The Fourth Affiliated Hospital of Harbin Medical University, Harbin, 150001 Heilongjiang China; 6 Department of Pediatrics, Perelman School of Medicine, University of Pennsylvania, Philadelphia, PA 19104 USA; 7 Division of Neurology, Perelman School of Medicine, The Children’s Hospital of Philadelphia, University of Pennsylvania, Philadelphia, PA 19104 USA; 8 Department of Pathology and Laboratory Medicine, Perelman School of Medicine, University of Pennsylvania, Philadelphia, PA 19104 USA; 9 Department of Pharmacology & Experimental Therapeutics, Boston University School of Medicine, Boston, MA 02142 USA; 10 Division of Human Genetics at Children’s Hospital of Philadelphia, Philadelphia, PA 19104 USA

**Keywords:** Bruton’s tyrosine kinase (BTK), B cells, Immunometabolism, Autoimmune diseases, Co-stimulatory molecules, Cytokines

## Abstract

Inhibition of Bruton’s Tyrosine Kinase (BTKi) is now viewed as a promising next-generation B-cell-targeting therapy for autoimmune diseases including multiple sclerosis (MS). Surprisingly little is known; however, about how BTKi influences MS disease-implicated functions of B cells. Here, we demonstrate that in addition to its expected impact on B-cell activation, BTKi attenuates B-cell:T-cell interactions via a novel mechanism involving modulation of B-cell metabolic pathways which, in turn, mediates an anti-inflammatory modulation of the B cells. In vitro, BTKi, as well as direct inhibition of B-cell mitochondrial respiration (but not glycolysis), limit the B-cell capacity to serve as APC to T cells. The role of metabolism in the regulation of human B-cell responses is confirmed when examining B cells of rare patients with mitochondrial respiratory chain mutations. We further demonstrate that both BTKi and metabolic modulation ex vivo can abrogate the aberrant activation and costimulatory molecule expression of B cells of untreated MS patients. Finally, as proof-of-principle in a Phase 1 study of healthy volunteers, we confirm that in vivo BTKi treatment reduces circulating B-cell mitochondrial respiration, diminishes their activation-induced expression of costimulatory molecules, and mediates an anti-inflammatory shift in the B-cell responses which is associated with an attenuation of T-cell pro-inflammatory responses. These data collectively elucidate a novel non-depleting mechanism by which BTKi mediates its effects on disease-implicated B-cell responses and reveals that modulating B-cell metabolism may be a viable therapeutic approach to target pro-inflammatory B cells.

**Supplementary Information:**

The online version contains supplementary material available at 10.1007/s00401-022-02411-w.

## Introduction

Inhibition of Bruton’s tyrosine kinase (BTK) using BTK inhibitors (BTKis) is being actively pursued as a novel non-depleting B-cell targeting approach in patients with multiple sclerosis (MS). To date, two phase II trials of BTKis in patients with MS have demonstrated an ability to limit relapsing MS disease activity [40, 45] and multiple small molecule BTKis are being actively pursued in different phases of MS trial development. Surprisingly little is actually known; however, about how inhibition of BTK impacts MS disease-relevant functions of human B cells. Most prior human work has considered the effects of BTKi on malignant cells and studies into the therapeutic mode of action of BTKi on non-malignant cells in autoimmune diseases including MS have largely focused on murine models [9, 20, 23, 28, 52].

BTK is expressed by B cells and innate cells, principally myeloid cells [19, 55]. In B cells, BTK is known to be an important downstream signaling molecule of the B-cell receptor and inhibitors of BTK were first used in humans to treat B-cell malignancies known to rely on BCR-mediated BTK signaling for survival [24]. Subsequent observations that BTK overexpression by B cells can manifest in generalized autoimmunity in mice [11, 29] and that circulating B cells of patients with rheumatoid arthritis and Sjogren’s disease over-express BTK [12], suggested that BTK inhibition may have therapeutic potential in human autoimmune diseases.

Human B-cell responses are now recognized as being context dependent such that different modes of activation can promote either pro-inflammatory or anti-inflammatory B-cell functions [30]. Mechanisms by which B cells contribute to autoimmune diseases can be both antibody-dependent and antibody-independent, and can differ across conditions. In MS, the contribution of B cells to relapsing disease activity is thought to principally reflect non-antibody dependent functions of the B cells in the periphery and, in particular, the capacity of activated B cells to aberrantly induce pro-inflammatory T-cell responses [7, 25, 31]. Whether and how inhibition of BTK impacts activation of human B cells under a range of stimulation conditions that they may encounter in vivo, and the consequences to B-cell:T-cell interactions considered relevant to MS, are unknown.

In the present study we demonstrate that in addition to its expected impact on B-cell activation, BTKi attenuates B-cell:T-cell interactions via a novel mechanism involving modulation of B-cell metabolic pathways which, in turn, mediates an anti-inflammatory modulation of the B cells. In vitro, effects of BTKi on B cells were not limited to BCR-mediated stimulation and were influenced by the mode of B-cell activation. Both BTKi and metabolic modulation could abrogate the aberrant activation and costimulatory molecule expression of B cells freshly isolated from untreated patients with MS, and the study of healthy volunteers participating in a Phase I trial of BTKi provided in vivo proof-of-principle that BTKi treatment reduces circulating B-cell mitochondrial respiration, diminishes their activation-induced expression of costimulatory molecules, and mediates an anti-inflammatory shift in the B-cell responses which is associated with an attenuation of T-cell pro-inflammatory responses. These data collectively elucidate a novel non-depleting mechanism by which BTKi mediates its effects on MS disease-implicated B-cell responses and reveals that modulating B-cell metabolism may be a viable therapeutic approach to target pro-inflammatory B cells.

## Materials and methods

### Study design, participants and samples

This is an experimental laboratory study performed with human samples. The study was designed to better understand the effects of BTKi on human B-cell functional responses. Healthy volunteers recruited among hospital staff, and MS patients recruited from the MS clinic (within 12 weeks of new MS diagnosis and never treated with immune therapy) at the University of Pennsylvania, provided written informed consent as approved by the Institutional Review Board (IRB). Participants with inherited mitochondrial mutations were recruited among family members of patients followed at the Children’s Hospital of Philadelphia with informed assent or consent, as appropriate, and as approved by the institutional IRB. The Phase I clinical trial of the highly selective BTKi (BIIB091) recruited healthy controls (NCT03943056) who provided informed consent through the Midlands IRB.

### Immune cell isolation and B-cell and T-cell culture

Peripheral blood mononuclear cells (PBMC) were separated by density centrifugation using Ficoll (GE health care). B cells were then isolated by CD19 magnetic bead following standard manufacturer’s protocols (Miltenyi biotec, Auburn, CA). Where indicated, CD4^+^ T cells were negatively selected using the CD4^+^ T-cell isolation kit II (Miltenyi biotec, Auburn, CA). The purity of each immune cell population was routinely confirmed as > 98%. All cells were cultured in serum-free x-vivo 10 media (Lonza). PBMC or B-cell-depleted PBMC (BD-PBMC) were plated in U bottom 96 plates at 2 × 10^5^. B cells were plated in U bottom 96-well plates at 3 × 10^5^/well in a total volume of 200ul per well. For co-culture assays, 1 × 10^5^ T cells were co-cultured with 2 × 10^5^ B cells in presence of various stimuli.

### BTK inhibitor selectivity

BIO-0556375 was developed as a highly selective BTK inhibitor with 100-fold greater selectivity to BTK when tested against a panel of 456 individual kinases using the DiscoverX KINOME*scan*™*.*

### B-cell and T-cell activation

Reagents used to activate or modulate B cells included soluble CD40L (1ug/ml, Enzo Life Sciences); Goat anti-human B-cell receptor F(ab′)2 fragment antibody (10ug/ml, Jackson ImmunoResearch); CpG DNA (1uM/ml, ODN2006, Invivogen); IL-4 (20 ng/ml, R&D system); IL-21 (20 ng/ml, R&D system); Rotenone (0.1uM, Sigma) and 2-DG (0.5 uM, Sigma). Reagents used to stimulate T cells included anti-CD3 (UCHT1, 1ug/ml); and Endotoxin-Free protein extracts from Heat Killed C. albicans (M15, Greer Labs, 1 ug/ml); or Heat-Killed S. aureus (TLRl-HKSA, Invivogen, 10^8^/ml). CFSE or Cell trace violet (CTV) were used, as per the manufacture’s protocols, when quantifying B-cell or T-cell proliferation. Briefly, isolated T cells were diluted in pre-warmed x-vivo medium at 1 × 10^6^ cells/ml with 1uM CFSE or 0.5 uM CTV slowly added to the cell suspension. Cells were then incubated at 37° in a water bath for 20 min, washed twice and then plated into 96 well U bottom plates.

### Flow cytometry

Annexin V and PI (BD Bioscience) were used to measure B-cell survival based on the manufacturer’s protocol. Immunophenotyping of B-cell and T-cell subsets, as well as T-cell proliferation and their cytokine expression using intracellular cytokine staining (ICS) were performed with antibodies targeting: CD3 (SK7), CD4 (RPA-T4), CD8 (RPA-T8), CD20 (2H7), CD80 (BB1), CD86 (2331(Fun-1)), IL-4 (8D4-8), IL-6 (MQ2-6A3), IL-10 (JEF3-19F1), TNFα (MAb11) and GM-CSF (BVD2-21C11), IL-17A (N49-653) and IFNγ (B27). All antibodies were obtained from BD Bioscience. To assess ICS, PMA (20 ng/ml, Sigma-Aldrich), Ionomycin (500 ng/ml, Sigma-Aldrich) and Golgi stop (Monensin, BD Bioscience) were added to cells 4–5 h before staining. To avoid potential confounding of dying/dead cells, we routinely used Live/Dead Aqua staining (Life technologies) to exclude dying/dead cells in all analyses except for viability assays, where we used Annexin V and PI. The Live/Dead reagent (1ul/1 × 10^6^ cells) was used at room temperature following the manufacturer’s protocol, after which cell-surface marker staining was performed. Cells were then fixed and permeabilized using fixation/permeabilization buffer (BD Bioscience). ICS antibodies (noted above) were added and incubated for 30 min on ice. Samples were then washed twice and analyzed by FACS Fortessa (BD Bioscience), stringently maintained by the flow cytometry core facility at the University of Pennsylvania.

### Enzyme-linked immunosorbent assay (ELISA)

Cytokine levels in culture supernatants were measured by OptEIA ELISA kit (GM-CSF and IFNγ, BD Bioscience), or ELISA READY-SET-GO Kit (IL-17A, IgG and IgA, ThermoFisher), following the manufacturers’ protocols. Briefly, ELISA plates were coated with capture antibody at least 12 h in advance. After another hour of blocking with blocking buffer (10% FCS in PBS), samples were added to the plate and incubated for 2 h at room temperature. Detection antibodies were then added and incubated for 1 h at room temperature. The plate was carefully washed with ELISA washing buffer (0.05% Tween 20, PBS) between each step. The color of the plate was eventually developed by TMB (BD Bioscience) and the reaction was stopped by 0.01 N H_2_SO_4_. Finally, the plates were read by a microplate reader (Varioskan, ThermoFisher Scientific).

### RNA analysis of gene expression by B cells

Total RNA was isolated from the differentially stimulated and treated B cells using the RNeasy Plus Micro Kit (Qiagen) according to the manufacturer’s protocol. RNA quantity and quality were assessed by TapeStation and Nanodrop. To calculate RNA integrity, samples were run on a Bioanalyzer (Agilent) with the RNA 6000 Pico kit (Agilent). All the samples that were sent for RNA sequencing have the RNA integrity number (RIN) above 7. RNA-seq was performed using a Novaseq6000 instrument (60 million reads/sample, 2 × 100 bp, illumina) with approximately 500 ng of total RNA. Raw reads were aligned to reference genome hg19 using STAR (Software: BasePair). Differentially expressed genes were calculated using DEseq2 (Software: BasePair). To adjust for multiple comparisons, the Benjamini–Hochberg procedure was applied to the data, with FDR < 0.05. PCA analysis was performed using BioVinci V1. The input data set for the PCA was the standardized gene expression data from each sample. The method for selecting PCs was based on parallel analysis that performs Monte Carlo simulations on random data of equal dimension to the input data and calculates eigenvalues for all resulting PCs. The number of simulations was 1000. Gene-set enrichment analysis was performed using GSEA software (A joint project by UC San Diego and Broad institute).

### Seahorse assays

B-cell mitochondrial respiration and glycolysis were detected by the XF Mitochondria stress test kit and Glycolytic rate assay kit (Agilent) following the manufacturers’ protocols. B cells were cultured under various conditions (as detailed in the figure legends) for 36 h. Cells were then harvested, counted and then re-plated in a Cell-tak (22.4 ug/ml, Corning) coated XF96 microplate with the same density across different conditions. To accelerate the attachment of cells to the plate, the plate was then centrifuged at 200 g for 1 min. The plate was then transferred to a 37 °C incubator not supplemented with CO_2_ for 50 ~ 60 min before analysis by Seahorse XF96 analyzer. Key components for the assay: XF media (non-buffered DMEM + 10 mM Glycose, 4 mM l-glutamine, and 2 mM sodium pyruvate), Oligomycin: 1uM, FCCP: 1uM, Rotenone/AA: 1uM and 2-DG: 200 mM.

### Statistics

All values are expressed as means ± SD or as individual data points, and *p* values are assessed as appropriate by student’s *t* test, one-way or two-way ANOVA, with Tukey post hoc test using Graphpad Prism version 9. Flow cytometry data were analyzed by FlowJo V10. Morpheus (Broad Institute, https://software.broadinstitute.org/morpheus) was used for heatmap generation and cluster analysis.

## Results

### BTKi limits B-cell activation, proliferation, and antibody production with minor impact on B-cell survival

**Fig. 1:**
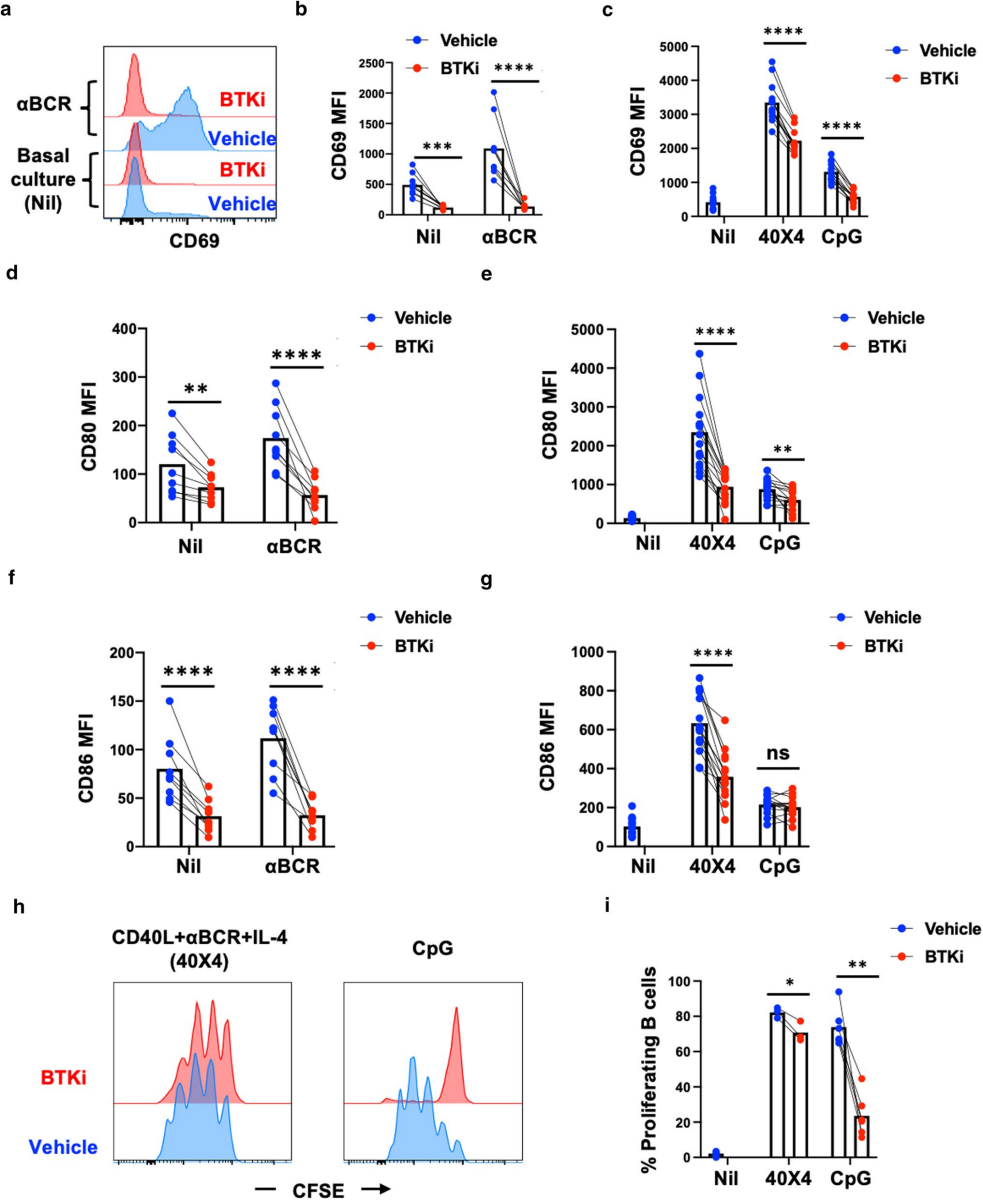
BTK inhibition decreases B-cell activation, co-stimulatory molecule expression and proliferation. Peripheral CD19^+^ B cells were isolated from venous blood of healthy donors (purity confirmation by flow cytometry routinely > 98%) and pre-treated with BTKi (1uM) or vehicle for 1 h, then left without stimulation (Nil) or stimulated under various conditions including activation through the BCR alone using BCR cross-linking antibody (αBCR), combined activation through the BCR together with CD40-ligand (CD40L) stimulation and IL-4 (40X4), or activation with CpG alone. CD69, CD80 and CD86 surface expression was measured on day 2 using flow cytometry and CFSE dilution was used to measure B-cell proliferation on day 5. BTKi treatment strongly decreases activation induced CD69 expression by B cells across the different stimulation conditions (*n* > 8) (**a**–**c**). CD80 expression is decreased with BTKi treatment across stimulation conditions (**d** and **e**), while CD86 expression is only reduced by BTKi under αBCR and CD40L + αBCR + IL-4 stimulation conditions (**f** and **g**, *n* = 12/13). **h** and **i** BTKi decreases B-cell proliferation, in particular upon CpG stimulation (*n* = 3/5). Repeated measure Two-way ANOVA, each line represents paired results from individual donors. *ns* not significant, *p < 0.05, **p < 0.01, ***p < 0.001 and ****p < 0.0001

To date, the effect of BTK inhibition (BTKi) on non-neoplastic B cells has largely been studied in mice, where it has been shown to limit signaling downstream of the B-cell receptor (BCR), thereby diminishing BCR-mediated activation [29, 49]. Using primary peripheral blood human B cells, we recapitulated and extended this observation showing that BTKi strongly limited BCR-mediated (αBCR) induction of the activation markers CD69 (Fig. 1a, b) and CD40 (Supplementary Fig. 1a), but also diminished expression of these activation markers under basal culture conditions (Nil), suggesting that the effects of BTKi on human B-cell responses may not be limited to consequences of BCR-mediated signaling. We, therefore, assessed the effects of BTKi on responses of human B cells under additional stimulation conditions. Those conditions are previously used as proxies to different modes of activation that B cells are thought to encounter in vivo [10, 27, 56]. We found that BTKi limited B-cell activation-induced upregulation of CD69 (Fig. 1c) and the co-stimulatory molecules CD80 (Fig. 1d, e) and CD86 (Fig. 1f, g), as well as B-cell proliferation assessed as CFSE dilution (Fig. 1h, i) across a range of stimulation conditions including stimulation with the TLR9 agonist CpG. Also of note, these BTKi effects, while having little or no impact on B-cell survival (Supplementary Fig. 1b), were all dose-dependent, with the magnitude and profile of these effects differing between stimulation conditions (Supplementary Fig. 2), indicating a context-dependent regulation of human B-cell function by BTKi.

### BTKi impacts B-cell:T-cell interactions

B cell contributions to MS pathophysiology are thought in part to reflect their capacity to interact with T cells, whether through elaboration of soluble factors, such as cytokines, or through expression of costimulatory molecules and potential as antigen presenting cells (APC) [25, 39]. Given the ability of BTKi to limit B-cell expression of costimulatory molecules, we next assessed the impact of BTKi on B-cell:T-cell interactions. We initially polyclonally stimulated either whole peripheral blood mononuclear cells (PBMC) or B-cell-depleted PBMC with anti-CD3 and anti-BCR, in presence of vehicle control or BTKi. Compared to the vehicle control, BTKi decreased polyclonal proliferation of both CD4^+^ T cells (Supplementary Fig. 3a) and CD8^+^ T cells (Supplementary Fig. 3b) and diminished their respective proinflammatory Th1 and Th17 (Supplementary Fig. 3c) and Tc1 (Supplementary Fig. 3d) cytokine responses. These effects were not seen in the B-cell-depleted PBMC, suggesting that the impact of BTKi on T-cell activation and cytokine expression was mediated through the B cells. In keeping with this, addition of BTKi directly to purified T cells did not affect T-cell proliferation or cytokine expression (Supplementary Fig. S4).

**Fig. 2:**
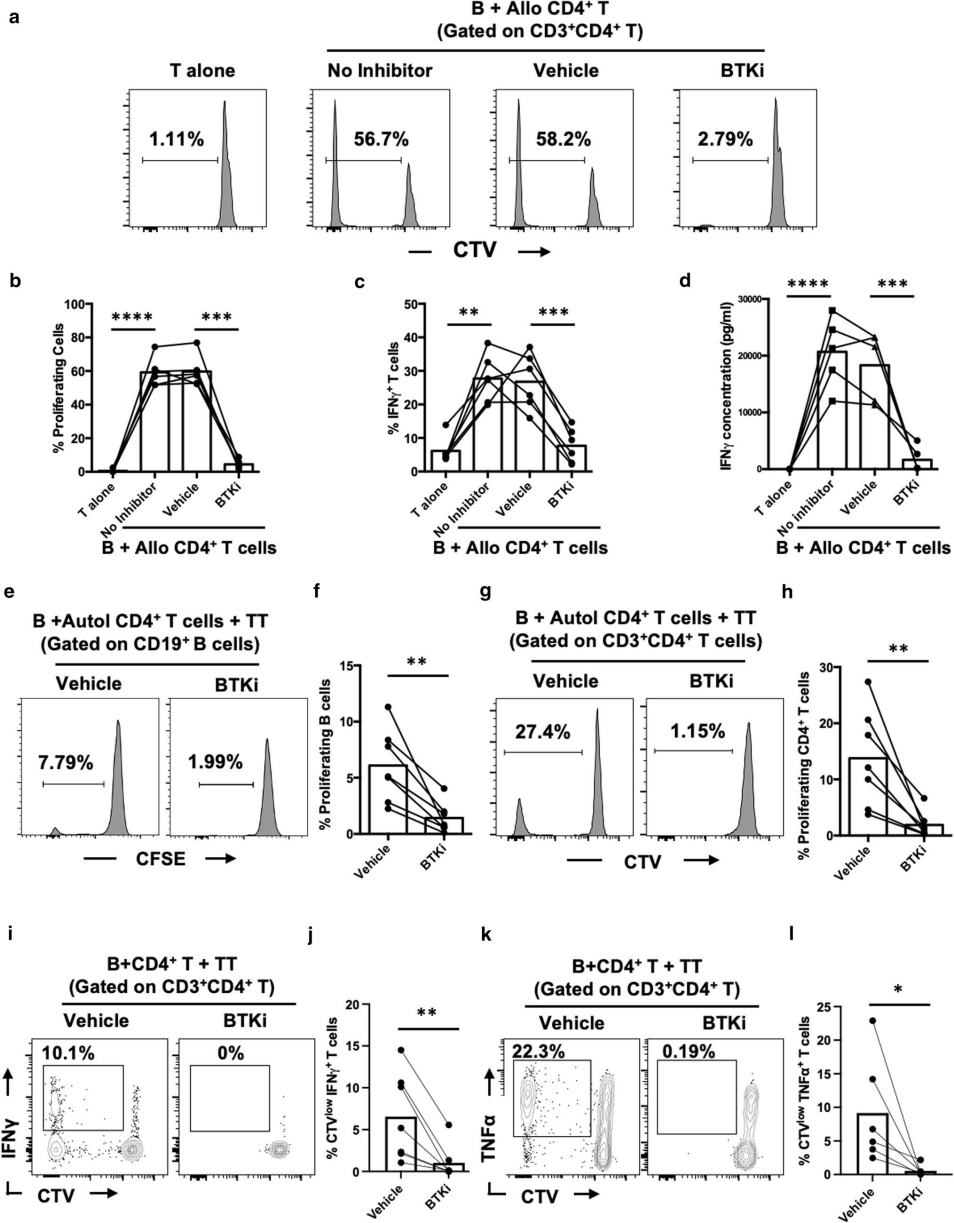
Inhibition of BTK diminishes allogenic and antigen-specific B-cell:T-cell interactions. BTKi or vehicle pre-treated human B cells were co-cultured with cell trace violet (CTV)-labelled allogenic CD4^+^ T cells for 7 days. BTKi treatment substantially diminishes allogenic T-cell responses (*n* = 5/6) (**a**–**d**). BTKi or vehicle pre-treated CFSE labeled human B cells were co-cultured with Cell trace violet (CTV) labelled CD4^+^ T cells in presence of Tetanus Toxoid (TT) for 12 days. Proliferation of B cells and CD4^+^ T cells was assessed as dilution of CFSE or CTV, respectively. T-cell cytokines (IFNγ and TNFα) were measured by FACS intracellular staining. BTK inhibition decreases both B-cell (**e** and **f**) and T-cell (**g** and **h**) proliferative responses to tetanus toxoid (*n* = 7) in the same co-culture system. In addition, BTKi decreases both IFNγ (**i** and **j**) and TNFα (**k** and **l**) expression by T cells (*n* = 6). **a**–**d** Repeated measure one way ANOVA; **e**–**l** paired *t* test. Repeated measure Two-way ANOVA, each line represents paired results from individual donors. *p < 0.05, **p < 0.01, ***p < 0.001 and ****p < 0.0001

We next assessed the effects of BTKi on antigen driven B-cell:T-cell interactions, first using an allogeneic system in which either vehicle- or BTKi-treated B cells were cultured with purified allogeneic CD4^+^ T cells. BTKi strongly decreased proliferation and cytokine expression and secretion by T cells responding to allogeneic B cells (Fig. 2a–d). Next, using our previously described autologous antigen-specific B-cell: T-cell co-culture system [32], we observed that BTKi significantly diminished the proliferation of both the B cells (Fig. 2e, f) and T cells (Fig. 2g, h), and limited expression of the T-cell pro-inflammatory cytokines IFNγ (Fig. 2i, j) and TNFα (Fig. 2k, l) in response to the recall vaccine-associated tetanus toxoid (TT), as well as to pathogen-associated antigens (Supplementary Fig. 5). Taken together, these results indicate that BTKi can impact cognate B-cell:T-cell interactions by directly diminishing B-cell responses and thereby indirectly limiting T-cell effector responses.

### BTKi modulates B-cell metabolic processes

**Fig. 3:**
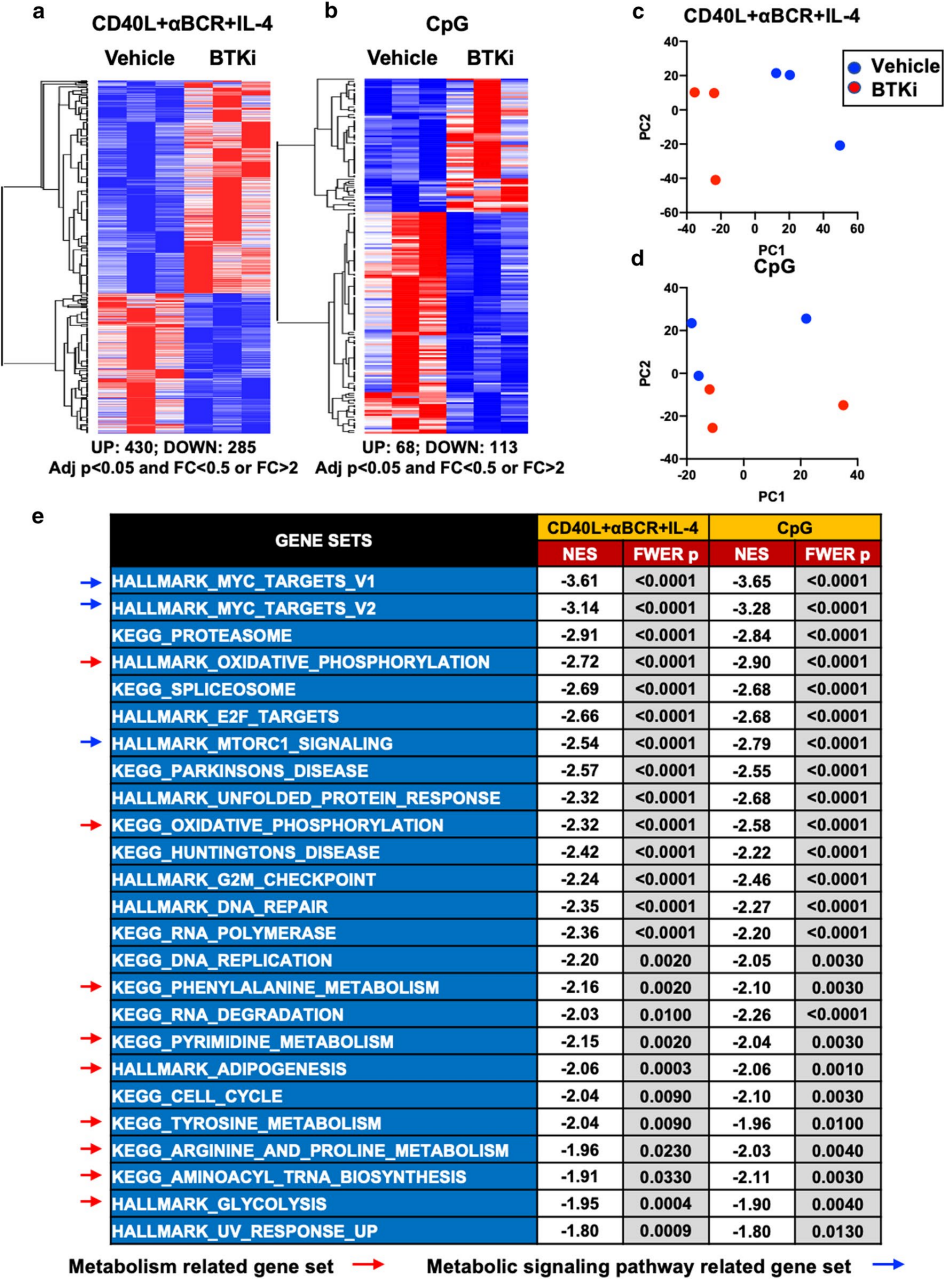
Transcriptomic changes induced by BTKi across different modes of B-cell activation. Purified healthy donor human B cells, pre-treated with either vehicle or BTKi for 30 min, were stimulated with either CD40L + αBCR + IL-4 or CpG for 18 h followed by bulk RNA-sequencing to test the impact of BTKi on activation-induced B-cell transcriptomic changes. Heatmaps together with hierarchical clustering (one minus spearman rank correlation) and principal component analyses were used to visualize the differentially expressed genes (**a**–**d**) with adjusted *p* value (Adj *p*) < 0.05; Fold change (FC) > 2 across different conditions (*n* = 3). Gene-set enrichment analysis (GSEA) against KEGG- and Hallmark-gene sets for those genes that were similarly impacted by BTKi across the stimulation conditions (**e**). As indicated by the negative NES (normalized enrichment score) values, 25 shared gene sets are inhibited in the BTKi condition relative to the vehicle control, among which nearly 50% (12/25) are implicated in either metabolic pathways (9/25, red arrows) or important signaling pathways that control metabolic processes (3/25, blue arrows)

**Fig. 4:**
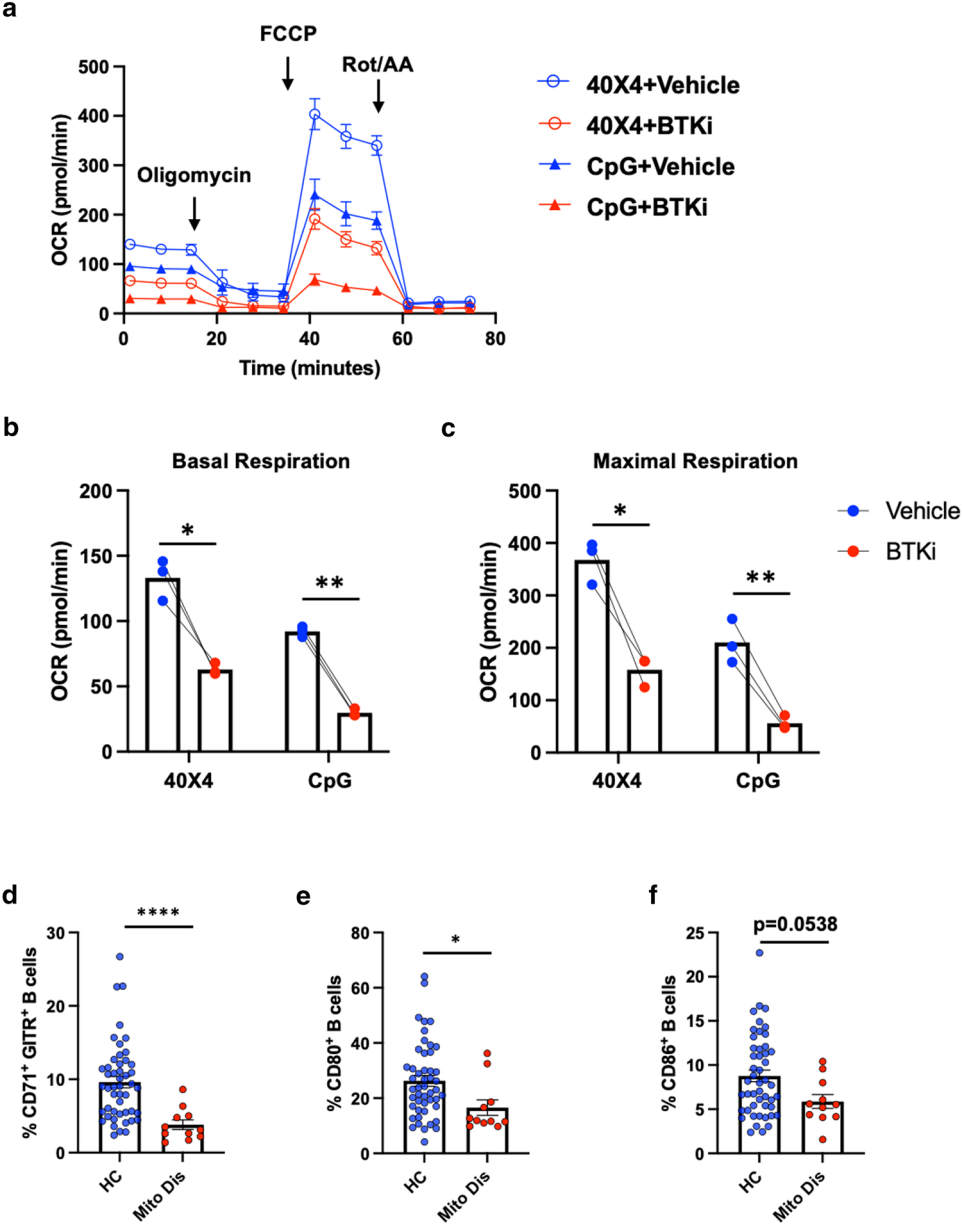
BTK inhibition down-modulates B-cell mitochondrial respiration and mitochondrial respiratory-chain mutations result in diminished B-cell activation and APC potential. Purified human peripheral B cells, pre-treated with either vehicle or BTKi for 30 min, were then stimulated with either CD40L + αBCR + IL-4 or CpG for 36 h. Seahorse mitochondrial stress testing was used detect mitochondrial-related metabolic changes of B cells. As illustrated in a representative oxygen consumption rate (OCR) curve of the seahorse mitochondrial stress assay (**a**), inhibition of BTK decreases both basal respiration (**b**) and maximal respiration (**c**) of B cells (*n* = 3). To assess the impact of mitochondrial respiratory chain mutations on human B cells, flow cytometry was applied to B cells purified ex vivo from patients with known mitochondrial complex I (ND3 or ND6) or V (USMG5) respiratory chain mutations (*n* = 11; see Table S1) and healthy controls (*n* = 48). Compared to healthy controls (HC), B cells of patients with mitochondrial respiratory chain mutations (Mito Dis) exhibited decreased activation profiles assessed as reduced frequencies of CD71^+^ and GITR^+^ B cells (d), and reduced expression of the co-stimulatory molecules CD80 and CD86 (**e** and **f**). **b** and **c** Repeated measure two-way ANOVA; Each line represents paired results from individual donors. **d**–**f** Non-parametric *t* test. *p < 0.05, **p < 0.01, and ****p < 0.0001

To investigate the potential mechanisms underlying the impact of BTKi on B-cell responses, we applied unbiased RNA-sequencing to either vehicle or BTKi-treated B cells across stimulation conditions (Fig. 3). Treatment with BTKi resulted in B-cell transcriptomic changes that partly differed across stimulation conditions (Fig. 3a–d). To isolate the BTKi effect, we focused on gene-set enrichment analysis (GSEA) of those genes that were similarly impacted by BTKi across the stimulation conditions. We observed that the majority of changes could be captured by 25 overlapping pathways (Fig. 3e). Notably, almost 50% (12 out of 25) of these appeared related to metabolic processes or signaling pathways that regulate cellular metabolism. This raised the possibility that BTK inhibition may regulate B-cell activation and function by modulating B-cell metabolic processes. Indeed, using the seahorse analyzer to monitor the metabolic activity of B cells exposed to BTKi or vehicle, we observed that BTKi decreased both basal and maximal B-cell mitochondrial respiration of activated B cells (Fig. 4a–c). BTK inhibition did not seem to impact mitochondrial respiration of human monocyte-derived macrophage, whether the myeloid cells were activated or left unstimulated (Supplementary Fig. 6), suggesting that the impact of BTK on cellular metabolism may be selective for B cells. As an in vivo biological proof-of-principle that dysfunction of mitochondrial respiration would manifest with abnormal activation profiles of human B cells, we were able to recruit a cohort of patients with known mitochondrial complex I (ND3 or ND6) or V (USMG5) respiratory chain mutations (Table. S1). We discovered that while the number of circulating B cells was not affected in these patients (Supplementary Fig. 7), their B cells exhibited substantially reduced expression of CD71 and GITR (Fig. 4d) reflecting less in vivo activation, as well as lower levels of the costimulatory molecules CD80 and CD86 (Fig. 4e, f). These data collectively indicate that mitochondrial respiration is involved in the regulation of human B-cell activation and co-stimulatory molecule expression.

### Inhibiting mitochondrial respiration recapitulates the effects of BTKi, and both can abrogate the aberrant activation and costimulatory molecule expression profile of MS patient B cells

**Fig. 5:**
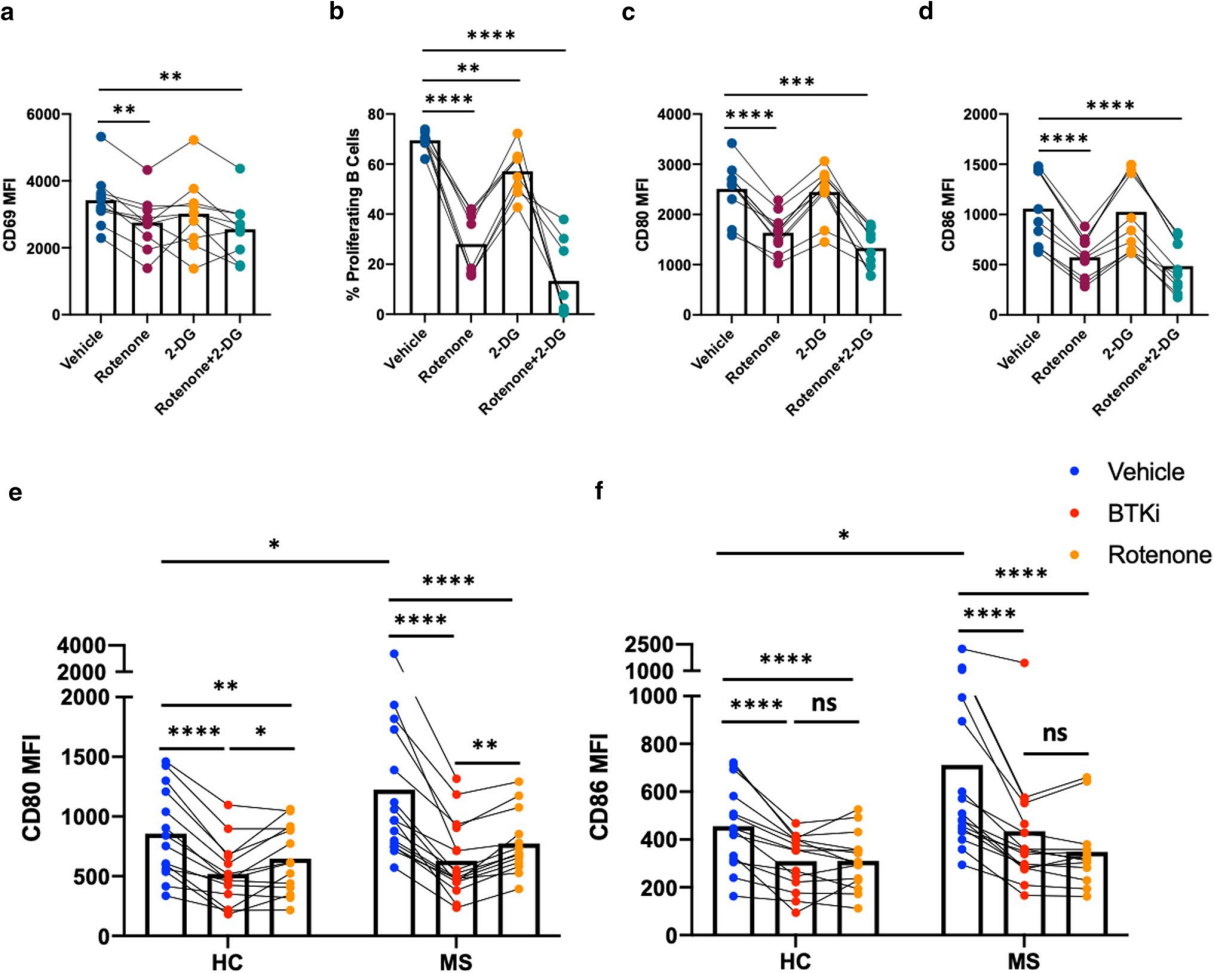
In vitro inhibition of mitochondrial respiration recapitulates the effects of BTKi on normal human B-cell activation and both inhibition of mitochondrial respiration and BTKi abrogate aberrant activation and costimulatory molecule expression of MS patient B cells. Purified healthy donor B cells were pre-treated with either vehicle, Rotenone (to inhibit mitochondrial respiration), 2-DG (to inhibit glycolysis), or the combination of Rotenone and 2-DG for 30 min, and then left unstimulated or stimulated with CD40L + αBCR + IL-4 for 2 days to assess expression of the activation marker CD69 and co-stimulatory molecules CD80, CD86; or for 5 days to assess proliferation by CFSE dilution, using flow cytometry. In the absence of stimulation (data not shown), average mean fluorescence intensities (MFIs) of B cell CD69, CD80 and CD86 expression in the ‘vehicle’ condition were approximately 1000, 75 and 125, respectively. Following stimulation which induced the expected proliferation and upregulation of activation and costimulatory molecules, inhibition of glycolysis was found to modestly limit B-cell proliferation (**b**) while having limited or no appreciable effect on B-cell activation (**a**) or activation-induced upregulation of costimulatory molecules (**c** and **d**, *n* = 10). In contrast, inhibiting mitochondrial respiration significantly decreased B-cell activation and proliferation, and limited the activation-induced upregulation of costimulatory molecules (**a**–**d**). To assess the effects of inhibiting mitochondrial respiration or BTK signaling on B-cell costimulatory molecule expression of MS patients, peripheral B cells were purified from either newly diagnosed (never-treated) MS patients, or healthy controls (HC) balanced for age and sex (Table S2 for participant demographics). The B cells were then treated with vehicle, BTKi or Rotenone and stimulated with CD40L + αBCR + IL-4 for 2 days. Expression of the costimulatory molecules CD80 (**e**) and CD86 (**f**) was measured by flow cytometry. Compared to HC, activated B cells of MS patients express abnormally higher levels of CD80 and CD86, and inhibition of BTK or inhibition of mitochondrial respiration with Rotenone, decrease the activation-induced B-cell expression of CD80 and CD86 by both HC and MS patient B cells, such that levels in MS patients are similar to those observed in HC (*n* = 13–16). **a**–**d** Repeated measure two-way ANOVA; **a**–**d** Mix-effects two-way ANOVA; Each line represents paired results from individual donors. *ns* not significant, *p < 0.05, **p < 0.01 ***p < 0.001 and ****p < 0.0001

We next tested how mitochondrial respiration and glycolysis may influence B-cell activation, proliferation, and co-stimulatory molecule expression. Using Rotenone to limit mitochondrial respiration and 2-DG to limit glycolysis (both at concentrations that did not diminish B-cell survival), we found that the in vitro inhibition of mitochondrial respiration (but not of glycolysis) results in decreased B-cell activation (Fig. 5a), while both mitochondrial respiration and glycolysis are involved in B-cell proliferation, though with a considerably greater reliance on mitochondrial respiration (Fig. 5b). We further found that mitochondrial respiration (but not glycolysis) is important for B-cell co-stimulatory molecule expression (Fig. 5c, d).

Since we observed that both inhibition of BTKi and inhibition of mitochondrial respiration limited costimulatory molecule expression of normal human B cells, and since patients with MS have been reported to harbor B cells that can contribute to exaggerated pro-inflammatory T-cell responses in part through aberrant expression of costimulatory molecules [8, 18, 32], we wished to assess the impact of BTKi or inhibition of mitochondrial respiration on costimulatory molecule expression by B cells isolated from patients with MS. To this end, we recruited newly diagnosed (never treated) MS patients as well as healthy controls (HC) balanced for age and sex (Table S2) and compared the effects of BTKi or inhibition of mitochondrial respiration on their B-cell activation. We first noted that B cells of MS patients exhibit abnormally increased activation-induced expression of the costimulatory molecules CD80 and CD86 under the vehicle control condition (Fig. 5e, f), extending prior reports [16, 18]. We further observed that treatment with BTKi, as well as inhibition of mitochondrial respiration by Rotenone, could each abrogate the aberrant activation-induced upregulation of co-stimulatory molecules expressed by MS patient B cells (Fig. 5e, f).

### BTK inhibition alters B-cell metabolism and costimulatory molecule expression partially through the PI3K/AKT/mTOR pathway

We considered that the ability of either BTKi or rotenone to limit B-cell costimulatory molecule expression could reflect either two independent pathways involved in costimulatory molecule regulation, or potentially the same pathway in which the effect of BTKi on costimulatory molecule expression is mediated through inhibition of mitochondrial respiration. In keeping with the possibility that the effect of BTKi is at least in part mediated through inhibition of mitochondrial respiration, we found that dual inhibition of both BTK and mitochondrial respiration resulted in little or no added inhibition (Supplementary Fig. 8), and sought to explore the potential mechanism involved. Since previous studies have shown that BTK interacts with the PI3K/AKT/mTOR pathway which, in turn, is known to control a range of cellular processes including metabolism [13, 17, 47, 48, 51], and since our bulk RNA-seq data indicated that BTKi treatment decreases expression of molecules associated with mTORC1 signaling, we speculated that BTK may regulate human B-cell metabolism through PI3K/AKT/mTOR. To test that possibility, we first measured phosphorylated AKT (pAKT) levels following anti-BCR stimulation with or without BTK inhibition. We observed that the inhibition of BTK significantly decreases BCR-induced phosphorylation of AKT while not appreciably affecting total AKT expression (Supplementary Fig. 9), suggesting that BTKi may suppress AKT activity and its down-stream pathway. We next found that inhibition of either PI3K, AKT or mTOR down-regulates expression of the B-cell co-stimulatory molecules CD80 and CD86, while also decreasing basal and maximal mitochondrial respiration, without affecting B-cell survival (Supplementary Fig. 10), all changes that mirror what we observed with BTK inhibition. Finally, there was no additive inhibitory effect when adding inhibition of either PI3K, AKT or mTOR to inhibition of BTK (Supplementary Fig. 11). Together, these findings suggest that BTK may regulate B-cell metabolism and co-stimulatory molecule expression through (or at least partially through) the PI3K/AKT/mTOR pathway.

### BTK inhibition alters B-cell metabolism and modulates B-cell and T-cell functional phenotypes in vivo

**Fig. 6:**
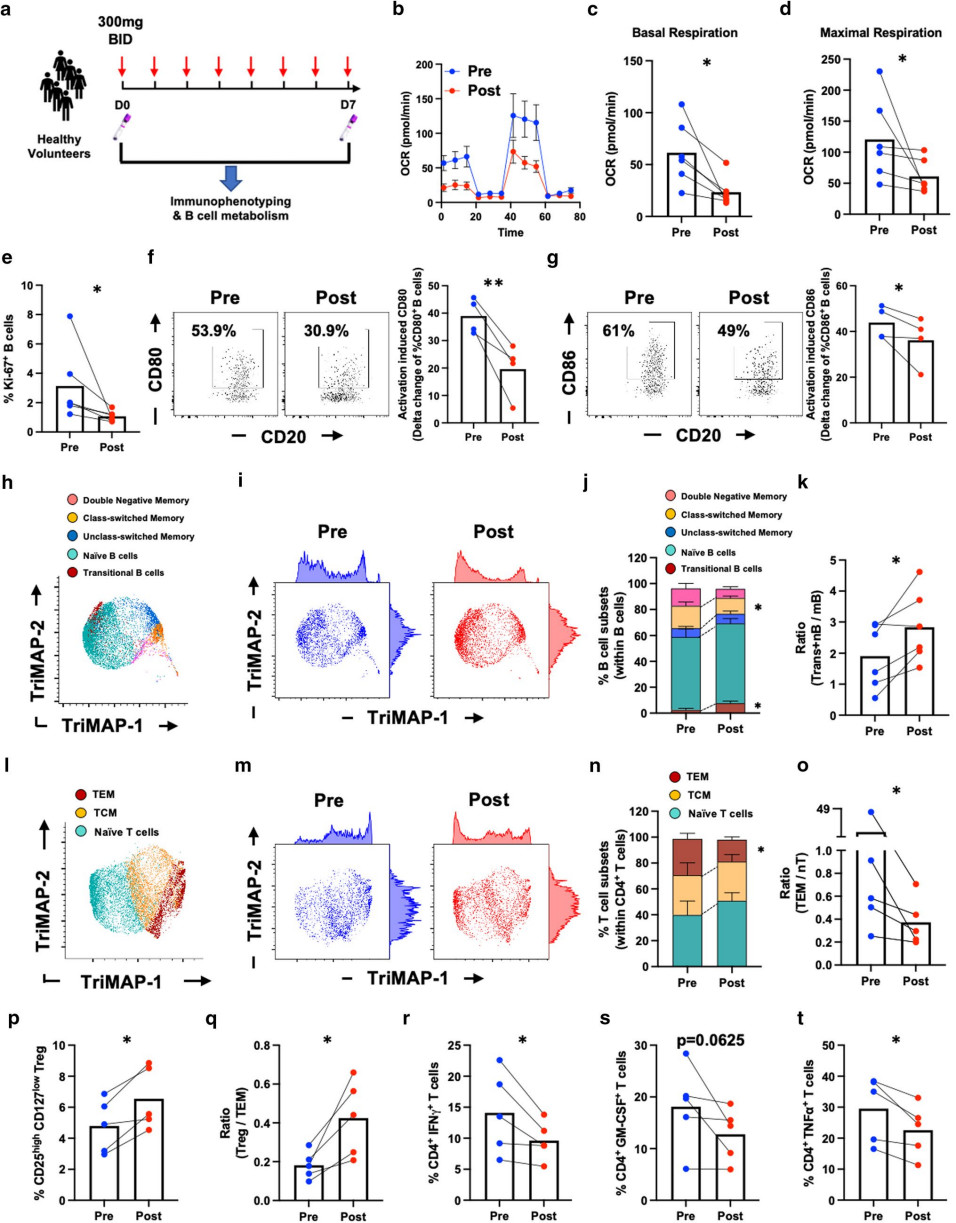
BTK inhibition alters B-cell metabolism and modulates B-cell and T-cell functional phenotypes in vivo. Healthy volunteers (*n* = 6) in a Phase I study were treated with 300 mg oral BTKi twice a day for 7 days (**a**). PBMC samples were collected and cryopreserved prior to treatment (D0) or day 7 on treatment (Post). B cells were purified from the PBMC and stimulated with CD40L + αBCR + IL-4 for 36 h, and seahorse mitostress assays were used to determine B-cell mitochondrial respiration. As illustrated in a representative oxygen consumption rate (OCR) curve (**b**), in vivo BTKi treatment significantly decreases both basal (**c**) and maximal (**d**) B-cell mitochondrial respiration. Multi-color flow cytometry panels were applied to PBMC from the same phase I trial participants to interrogate the phenotypic and functional profiles of both B cells and T cells. BTKi treatment substantially limits the frequency of proliferating Ki-67^+^ circulating B cells (**e**) and diminishes their activation-induced CD80 and CD86 expression (**f** and **g**). TriMAP dimensionality reduction was applied to the B-cell immunophenotyping data to visualize B-cell subset composition (**h** and **i**). In vivo BTKi treatment increases transitional and naïve B-cell frequencies while decreasing frequencies of circulating class-switched memory B cells (**j**), which results in an increased ratio of antigen inexperienced B-cell subsets (transitional + naïve B cells) to memory B cells (**k**). TriMAP dimensionality reduction applied to T-cell immunophenotyping (**l** and **m**) reveals that effector memory T cells are preferentially decreased by BTKi treatment (n), resulting in decreased ratios of effector memory (TEM) to naïve (nT) T cells (**o**). In contrast, the frequency of phenotypically defined regulatory T cells (Treg) increases (**p**), which together results in an anti-inflammatory shift of the balance of Treg and TEM T-cell subsets (**q**). In keeping with these phenotypic changes, in vivo BTKi treatment is found to diminish activation-induced pro-inflammatory cytokine-expressing T cells (**r**–**t**). **e**–**l** Paired *t* test. Each line represents paired results from individual donors. *p < 0.05

Together, our in vitro findings indicated that inhibition of BTK results in reduced B-cell mitochondrial respiration and decreases the capacity of B cells to stimulate and activate T cells. To test whether these effects of BTKi can occur in vivo in humans, we were able to take advantage of PBMC samples obtained as part of the phase I healthy control clinical trial of the highly selective oral BTK inhibitor (BIIB091) [4]. Using samples obtained both prior to, and after 7 days of daily treatment (Fig. 6a), we first confirmed that BTK inhibition reduced B-cell mitochondrial respiration in vivo including both basal respiration and maximal respiration (Fig. 6b–d). In addition, in keeping with our in vitro findings, B cells isolated from these BTKi treated individuals exhibited decreased frequencies of proliferating (Ki-67^+^) B cells (Fig. 6e), as well as diminished activation-induced upregulation of the costimulatory molecules CD80 and CD86 (Fig. 6f, g). Of note, these changes in B-cell functional profiles were associated with changes in the composition of circulating B-cell subsets: while transitional and naïve B cells were increased in frequency, the percentage of memory B cells was reduced by the in vivo BTKi treatment (Fig. 6 h–k). Finally, we observed that the in vivo BTKi treatment decreased the proportion of effector memory (TEM, CD45RA^−^ CCR7^−^) CD4^+^ T cells (Fig. 6l–o), while the frequency of phenotypically defined regulatory T cells (Treg, CD25^high^ CD127^−^) appeared increased (Fig. 6p), resulting in a substantial increase in the Treg/TEM ratio (Fig. 6q), consistent with an in vivo anti-inflammatory shift of T-cell subsets. In keeping with this, we observed that frequencies of circulating T cells expressing pro-inflammatory cytokines (including IFNγ, GM-CSF and TNFα) were all reduced in the individuals treated with BTKi (Fig. 6r–t). Collectively our data indicate that BTKi modulates human B-cell mitochondrial metabolism and limits pro-inflammatory responses of both B cells and T cells, in vitro and in vivo.

## Discussion

In addition to providing insights into the impact of BTK inhibition (BTKi) on responses of B cells and B-cell:T-cell interactions of potential relevance to the therapeutic mechanism of action of BTKi in MS, our study reveals a novel mechanism involving metabolic regulation of B-cell effector functions. We started by assessing the in vitro impact of BTKi on a range of human B-cell responses implicated in MS, including activation, proliferation and APC function. BTK inhibition results in the downregulation of both resting and activation-induced co-stimulatory molecule expression by human B cells which, in turn, influences their capacity to promote antigen specific pro-inflammatory T-cell responses. Notably, the effect of BTKi in down-regulating pro-inflammatory B-cell responses is associated with its ability to modulate B-cell mitochondrial respiration, without compromising cellular viability. We demonstrate that mitochondrial respiratory chain mutations in humans result in corresponding phenotypic and functional changes of their B cells in vivo and show that BTKi (or inhibition of respiration) can essentially abrogate the abnormal proinflammatory profile of untreated MS patient B cells ex vivo. Finally, we provide proof-of-principle of the in vivo impact of BTKi on human B-cell metabolism and its ability to limit pro-inflammatory responses of both B cells and T cells in a Phase I cohort of healthy controls.

The field of immunometabolism initially described roles of metabolism in the regulation of macrophage and T-cell functions [5, 54] with more recent animal work suggesting that cellular metabolism also plays a role in responses of germinal center B cells [26, 57]. In these studies, modulation of cellular metabolism both in vitro and in vivo (using either chemical inhibitors or specific knock-out mice) did not substantially impact the viability or survival of the immune cells. We extend these findings with the observations that patients with mitochondrial complex deficiency or healthy individuals exposed to BTKi, exhibit diminished B-cell mitochondrial respiration and modulated effector responses, without decreased viability or loss of major circulating cell subsets. Our finding, both in vitro and in vivo*,* that inhibition of BTK (and associated inhibition of mitochondrial respiration) does not significantly impact survival of normal B cells, points to fundamental differences in the roles of BTK and metabolic regulation of normal B cells as compared to neoplastic B cells, where BTKi is known to impact cell survival [50]. Indeed, in healthy individuals and in patients with MS, rather than decreasing B-cell counts, treatment with BTKi appears to transiently increase B-cell counts [4, 41]. We further demonstrate that cellular metabolic pathways are differentially involved in distinct responses of human B cells, while glycolysis contributes to proliferative responses, mitochondrial respiration appears more important for B-cell activation, and particularly for activation-induced upregulation of co-stimulatory molecules.

The contribution of B cells to the development of new MS relapsing disease activity which has been underscored by the success of B-cell targeting anti-CD20 therapies, is largely attributed to antibody-independent functions of B cells in the periphery, including their capacity to activate and serve as APC to pro-inflammatory T cells [6, 25]. Prior studies have shown that circulating B cells of MS patients express abnormally increased levels of the T-cell costimulatory molecules CD80 and CD86 [16, 18]. In addition to extending this finding by showing abnormal activation-induced upregulation of CD80 and CD86 by B cells of MS patients, we demonstrate that inhibition of either BTK or of mitochondrial respiration can abrogate this aberrant costimulatory molecule upregulation by the patient B cells. We further show that BTK inhibition effectively limits human B-cell:T-cell interactions including the capacity of B cells to function as APC and mediate antigen-specific pro-inflammatory T-cell responses. These observations complement elegant recent animal model work showing that treatment with BTKi decreases B-cell costimulatory molecule expression and limits B-cell APC function in experimental autoimmune encephalomyelitis, the commonly used animal model of MS [52]. Our findings further suggest that BTKi not only limits activation of B cells and their resultant capacity to activate T cells but may also shift the balance of pro- and anti-inflammatory B-cell responses. In our small cohort of healthy individuals exposed to BTKi in vivo, in addition to limiting B-cell mitochondrial respiration and diminishing their activation and costimulatory molecule expression, treatment resulted in an increased proportion of circulating transitional B cells, while the proportion of memory B cells decreased, consistent with the possibility that these B-cell subsets are differentially affected by BTKi. Since in MS regulatory functions have been attributed to transitional and naïve B cells, while the memory B-cell pool is considered to harbor B cells responsible for the aberrant activation of T cells, the overall effects we observe of BTKi on phenotypic and functional properties of B cells suggest that treatment with BTKi both limits activation and mediates a pro-inflammatory to anti-inflammatory shift in circulating B-cell responses.

While we focus here on effects of BTKi on peripheral B-cell responses and B-cell:T-cell interactions that are thought to be involved in the development of new MS relapses, BTK is also expressed on myeloid cells and the known capacity of BTKi to limit myeloid cell activation (with the potential to limit consequent T-cell activation) may also be relevant to the therapeutic mode of action of BTKi on MS relapses. Our observation that BTKi has little direct effect on T cells in vitro, yet diminishes T-cell pro-inflammatory cytokine responses in vivo, could reflect indirect effects of BTKi on T cells, mediated by either B cells, myeloid cells or a combination of both. Intriguingly, and similar to its in vivo effects on B cells, BTKi treatment not only diminished the pro-inflammatory activation of T cells, but also appeared to differentially impact T-cell subsets, resulting in decreased proportions of effector memory to naïve T cells, and increased proportions of phenotypically defined regulatory T cells to effector memory T cells. Of interest for future studies will be to confirm the in vivo effects of BTKi on the different subsets of B cells, T cells and myeloid cell in patients with MS, as well as elucidate whether B cells or myeloid cells are the main mediators of BTKi’s ability to modulate disease-relevant T-cell responses in vivo.

The development of CNS-penetrant inhibitors of BTK has raised the exciting prospect that such treatment could impact not only peripheral immune cells involved in MS relapses, but also elements of CNS-compartmentalized inflammation thought to be involved in non-relapsing progressive disease [7, 22, 35, 38]. Among potentially relevant BTKi targets for impacting progressive disease in MS are B-cell rich meningeal immune-cell infiltrates which also contain myeloid cells, T cells and stromal cells [53] and are associated with adjacent sites of subpial cortical demyelination and neuronal loss, linked with progressive disease [22, 35, 53]. A working hypothesis in the field is that chronic immune activation within the meninges may result in release of toxic soluble factors, possibly by B cells [33, 34, 42], which might contribute to the subpial cortical injury and injury of periventricular structures, such as the thalamus, which is also considered an important pathologic hallmark of progressive disease [3, 36]. It is attractive to speculate that a CNS-penetrant BTKi harboring the capacities that we demonstrate here to inhibit human B-cell mitochondrial respiration and activation, limit the interactions between B cells and T cells, and possibly mediate anti-inflammatory shifts in their profiles, might meaningfully interfere with cascades of immune activation and injury involved in progressive MS disease biology. An elegant proof-of-principle animal model study incorporating high-field MRI recently showed that BTKi treatment could effectively reduce meningeal inflammation in EAE [9] and substantiating such an effect in patients with MS is eagerly awaited.

Even if CNS-penetrant BTKi treatment indeed proves effective at limiting progressive MS, establishing that such an outcome is mediated through BTKi effects on CNS B cells will not be straightforward, since BTK is also expressed by infiltrating macrophage and resident tissue microglia. Microglial activation is recognized as a prominent feature of both the subpial and deep gray matter demyelinating lesions (noted above to be involved in progressive disease) and activated microglia are also implicated in a subset of perivascular MS lesions referred to as slowly expanding or slowly evolving lesions (SELs)—thought to represent chronic active lesions in which progressive tissue injury is propagated [1, 2, 14, 15]. Indeed, the potential for BTKi to inhibit activation of resident CNS microglia and thereby diminish their capacity to propagate CNS inflammatory injury has also been recently demonstrated in animals [43]. Whether considering microglia or B cells as BTKi targets of interest, it is also important to keep in mind that the actual contributions of these cells within the MS CNS are not fully elucidated and may well be heterogenous. Microglia may play pro-inflammatory and injurious roles but are also actively involved in maintaining tissue homeostasis and can play anti-inflammatory and potentially reparative roles in the injured CNS [21, 37, 58]. While our current B cell focused study highlights a mechanism by which BTKi may limit B-cell pro-inflammatory contributions in MS, mounting evidence also indicates that anti-inflammatory B cells and/or plasma cells can traffic into the inflamed CNS, where they may exert important anti-inflammatory, and potentially tissue protective, effects [44, 46].

In summary, our investigation of the impact of BTKi on human B cells reveals a novel mechanism by which BTK signaling promotes pro-inflammatory B-cell functions. We demonstrate that cellular metabolic pathways are involved in distinct functions of human B cells and highlight targeting of cellular metabolism, and particularly inhibition of mitochondrial respiration, as an approach for limiting pro-inflammatory activation of both B cells and T cells, and as a potential contributor to the therapeutic effects of BTK inhibition in MS. While the potential for BTKi to target B cells (as well as myeloid cells) both in the periphery and within the CNS compartment of patients offers the exciting prospect of addressing both relapsing and progressive disease mechanisms, there remain important knowledge gaps regarding how BTKi treatment will impact functionally diverse populations of these cells that may have contrasting (beneficial versus detrimental) effects. Incorporating novel imaging techniques and cutting-edge cell-based biomarker studies of blood and CSF into ongoing and future BTKi clinical trials in MS could help to more fully define the impact of BTKi treatment on the diverse functional response profiles of immune cells, and potentially help elucidate (and distinguish) their contributions to both relapsing and progressive disease mechanisms.

## Supplementary Information

Below is the link to the electronic supplementary material.

Supplementary file1 (PDF 1471 KB)

## Data Availability

All data are available in the main text or the supplementary materials.
