# Supplementary material for: BTK inhibition limits B-cell–T-cell interaction through modulation of B-cell metabolism: implications for multiple sclerosis therapy
Source: Acta Neuropathol. 2022 Mar 18;143(4):505–21. doi: 10.1007/s00401-022-02411-w (PMC8960592; doi:10.1007/s00401-022-02411-w)
Supplement: Supplementary file 1 — Supplementary file1 (PDF 1471 KB) [file 401_2022_2411_MOESM1_ESM.pdf]

Supplementary Fig. 1

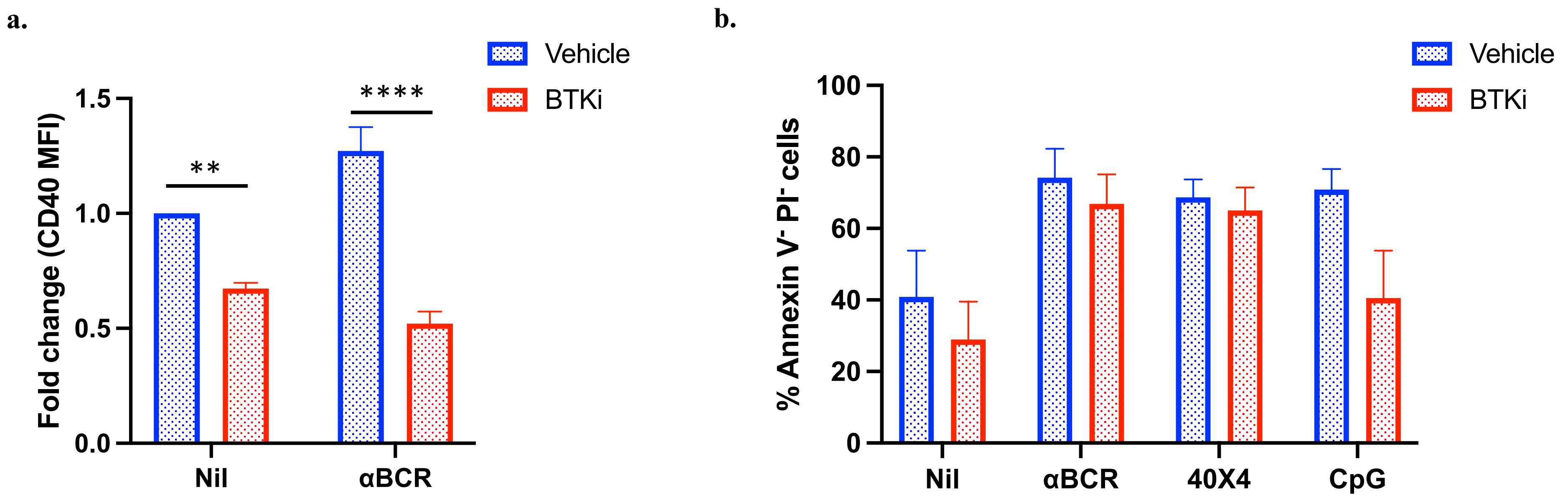

Supplementary Fig. 1. BTK inhibition decreases B-cell activation with limited impact on B-cell survival. Peripheral CD19^{+} B cells from healthy donors were pre-treated with BTKi or vehicle for 1 hour then cultured without stimulation (Nil) or with the addition of BCR cross-linking antibody (αBCR) for 48hrs. The expression of CD40 on live B cells was measured by flow cytometry. **(a).** BTKi decreases CD40 expression by B cells under both basal culture conditions and when activated with BCR cross-linking. **(b)** PI and Annexin V were used to test B-cell survival. Live B cells were defined as PI and Annexin V double-negative cells (n=7). **p<0.01 and ****p<0.0001.

Supplementary Fig. 2

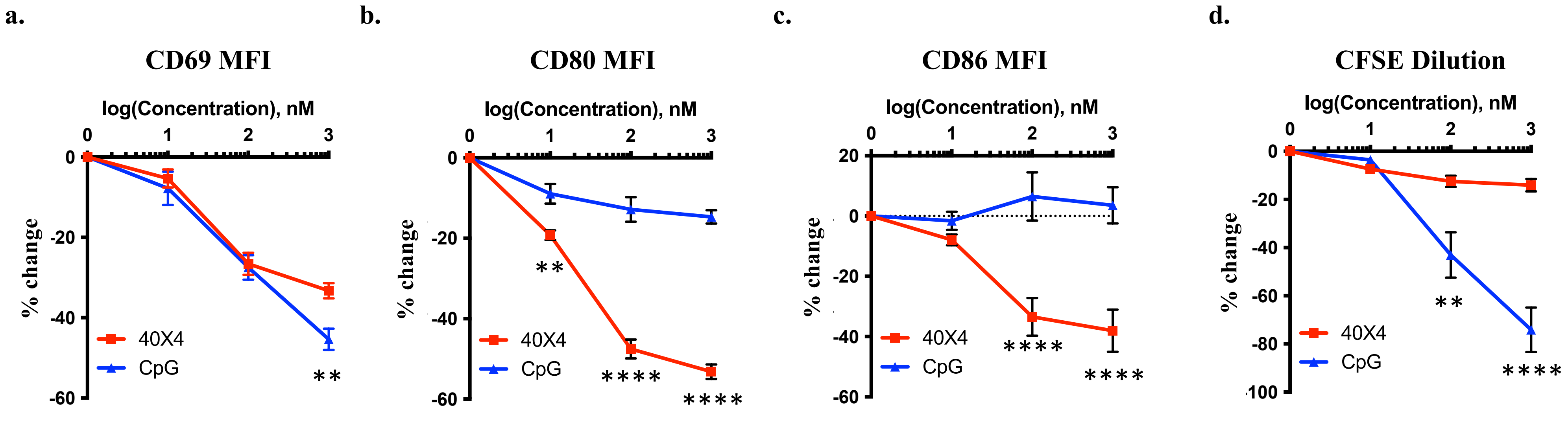

Supplementary Fig. 2. BTK inhibition mediates dose-dependent decreases in B-cell activation, co-stimulatory molecule expression and proliferation. Peripheral CD19$^+$ B cells from healthy donors were pre-treated with various doses of BTKi or vehicle for 1 hour before stimulation with either CD40L+αBCR+IL-4 (40X4) or CpG. CD69, CD80 and CD86 surface expression was measured on day 2 using flow cytometry and CFSE dilution was used to measure B-cell proliferation on day 5. **(a).** BTKi strongly decreases activation induced CD69 expression by B cells in a dose-dependent manner (n=5). **(b)** BTKi mediates dose-dependent decreases in CD80 expression across stimulation conditions; while **(c)** reducing CD86 expression only under the CD40L+αBCR+IL-4 stimulation conditions (n=5). **(d)** BTKi decreases B-cell proliferation, in particular upon CpG stimulation (n=3). **p<0.01 and ****p<0.0001.

Supplementary Fig. 3

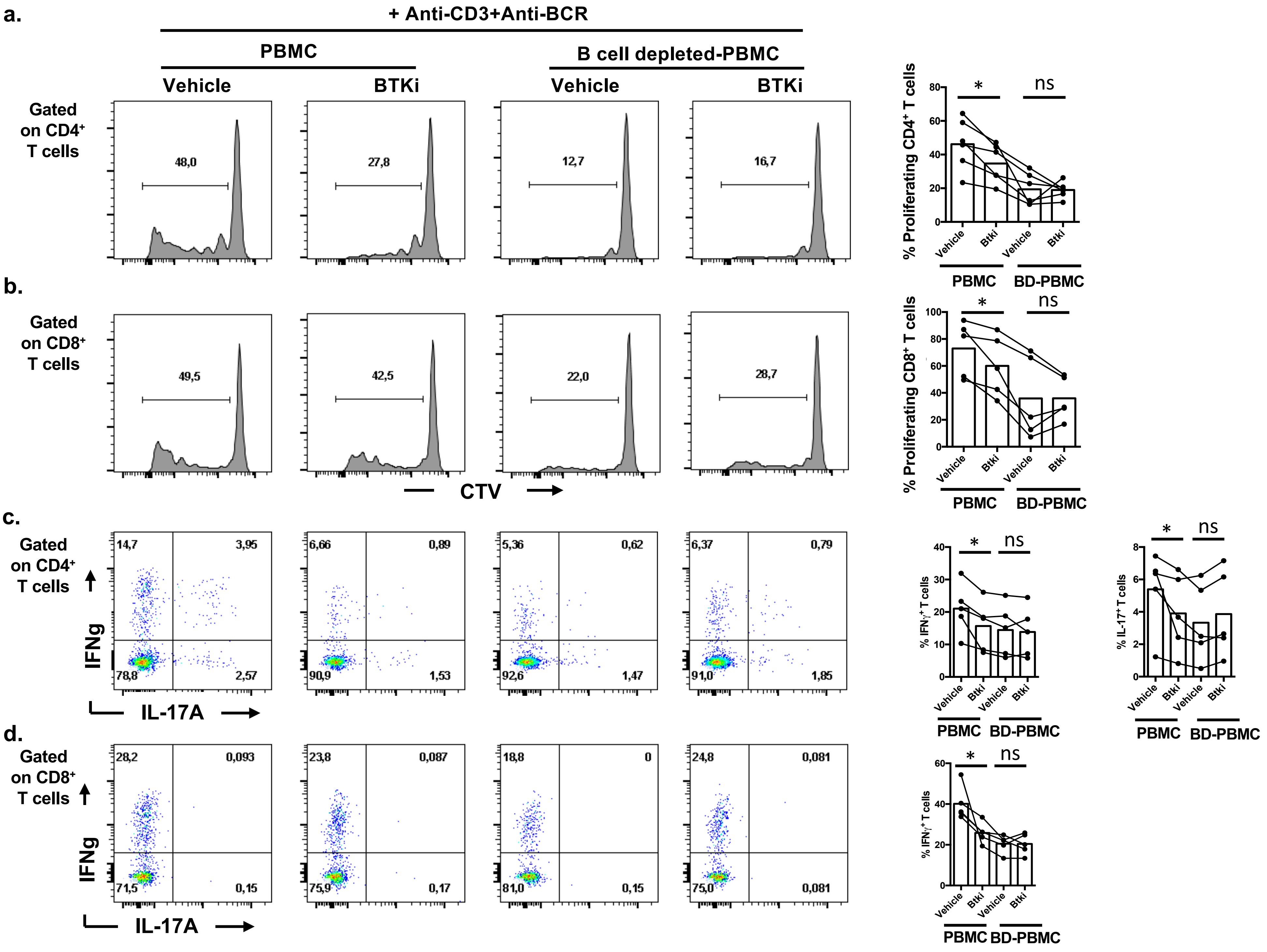

Supplementary Fig. 3. BTKi decreases T-cell responses within PBMC through B cells.

Cell trace violet (CTV) labelled healthy donor human peripheral blood mononuclear cells (PBMC), or B-cell depleted PBMC (BD-PBMC) were stimulated with anti-CD3 and anti-BCR in presence of either vehicle or BTKi. T-cell subset proliferation was measured by the dilution of CTV. T-cell cytokine expression was measured by FACS and intracellular cytokine staining (ICS). BTKi decreases both $CD4^+$ **(a)** and $CD8^+$ **(b)** T-cell proliferation and cytokine expression **(c and d)** within PBMC, but not within B-cell depleted PBMC. (n=5/6). ns: not significant, *$p<0.05$.

Supplementary Fig. 4

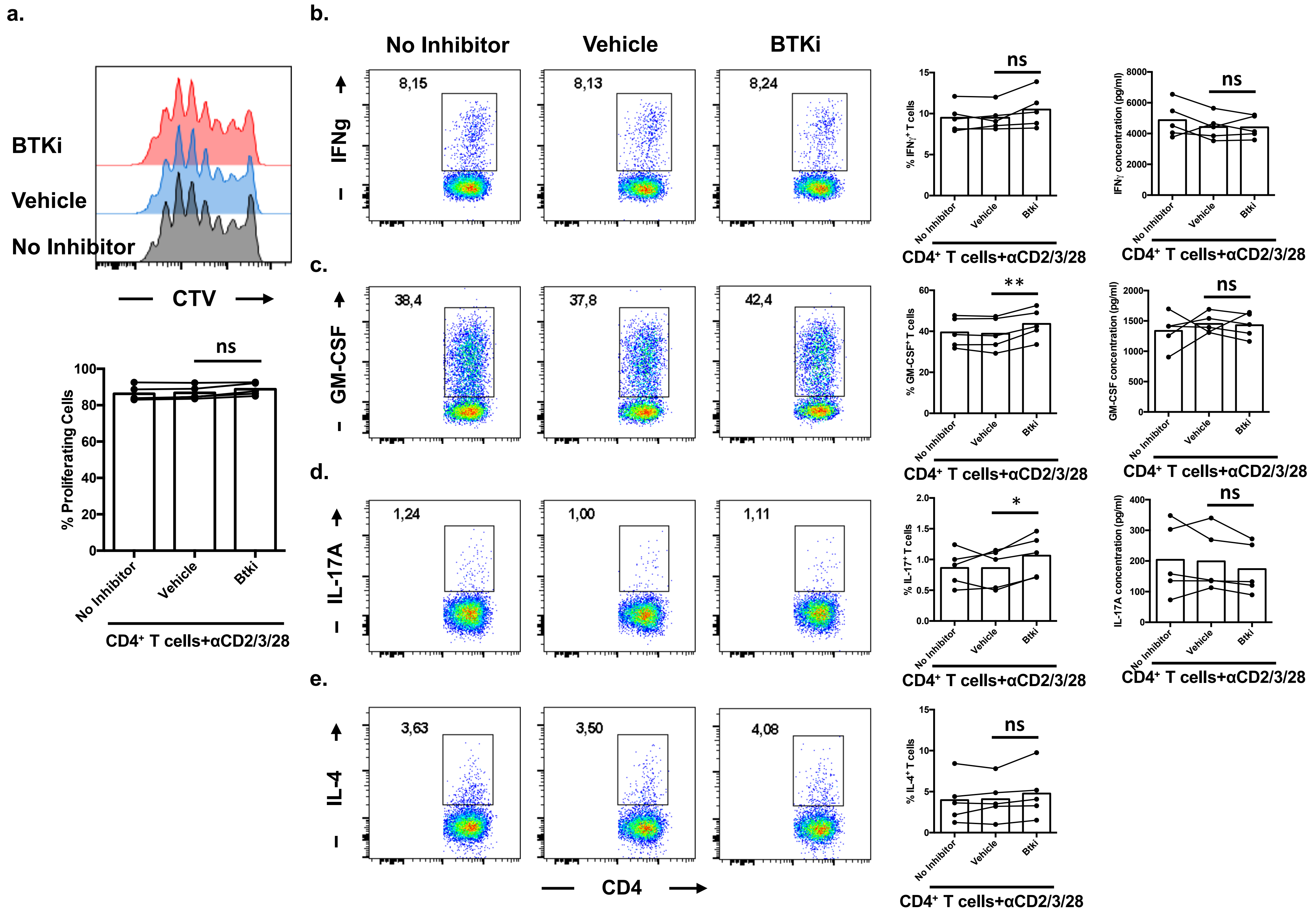

Supplementary Fig. 4. Direct exposure to BTKi does not influence T cell responses.

$CD4^+$ T cells were purified from healthy donor human peripheral blood mononuclear cells (PBMC) with purity confirmation by flow cytometry (routinely > 98%). Cell trace violet (CTV) labelled $CD4^+$ T cells were stimulated with anti-CD2/CD3/CD28 (αCD2/3/28) in presence of either vehicle or BTKi. T-cell proliferation was measured by the dilution of CTV. T cell expression of cytokines (IFNγ, GM-CSF, IL-17A and IL-4) was measured by FACS and intracellular cytokine staining (ICS), and cytokine secretion was quantified by ELISA. The addition of BTKi to purified $CD4^+$ T-cells did not alter activation induced T cell proliferation **(a)** or cytokine production **(b~e)**. (n=5). ns: not significant, $*p<0.05$.

Supplementary Fig. 5

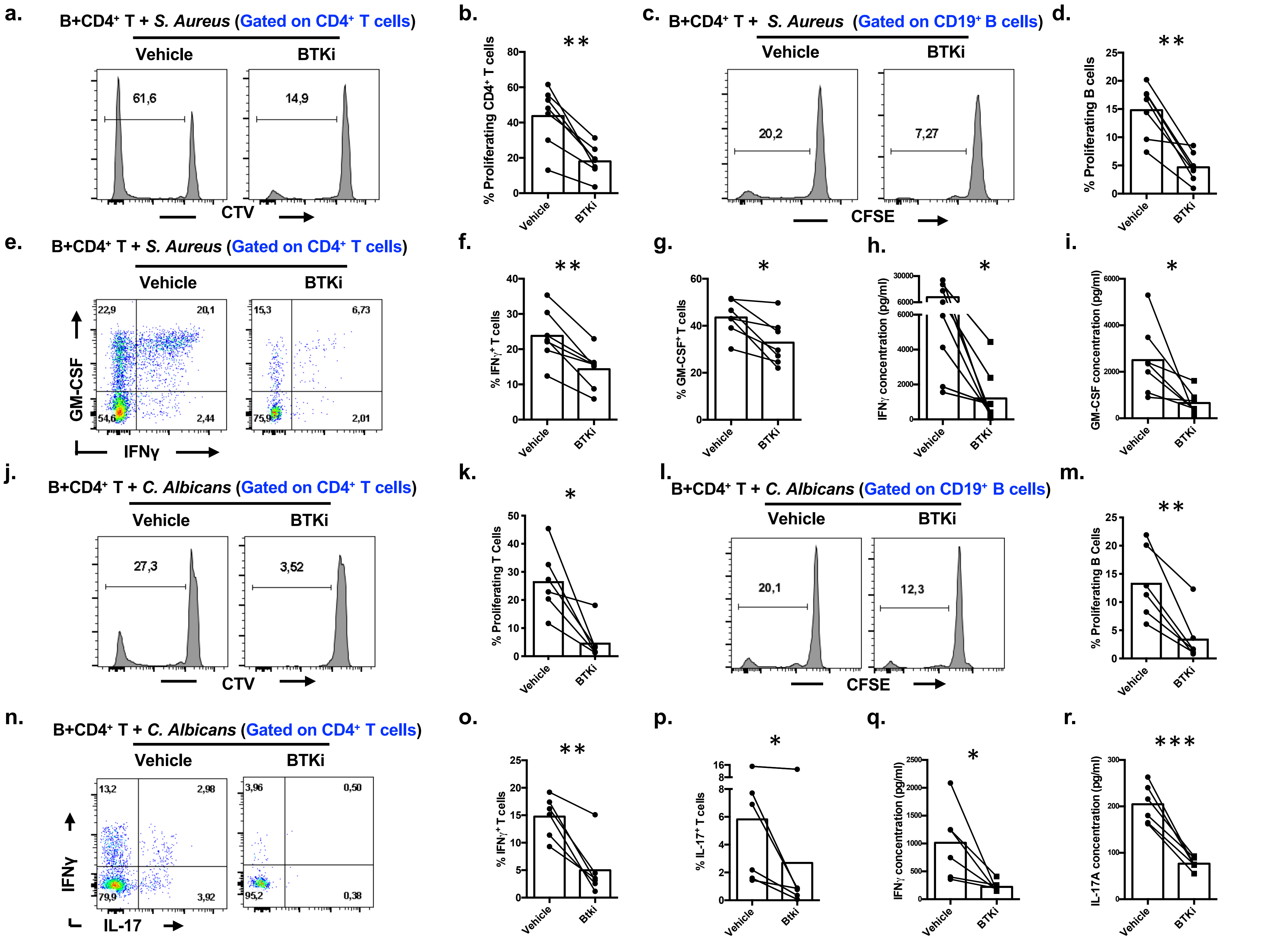

Supplementary Fig. 5. Inhibition of BTK diminishes *pathogen*-associated B-cell:T-cell interactions.

BTKi or vehicle pre-treated CFSE labeled purified human B cells were co-cultured with Cell trace violet (CTV) labelled $CD4^+$ T cells in presence of either heat-killed *Staph. Aureus* (HKSA) **(a-i)** or heat-killed *Candida Albicans* **(j-r)** for 12 days. $CD4^+$ T-cell and B-cell proliferation was traced by dilution of CTV or CFSE respectively. T cell cytokines (IFNγ, GM-CSF and IL-17) were measured by FACS and ELISA. The addition of BTKi limits B-cell mediated HKSA-specific proliferation of T cells **(a and b,** n=7**)** as well as HKSA antigen-associated B-cell proliferation in the same co-culture system (**c and d**). BTKi also decreases the HKSA-reactive pro-inflammatory T cell cytokine (IFNγ and GM-CSF) responses (**e-i**, n=7). Similarly, BTKi limits *Candida Albicans* specific proliferation of T cells **(j and k,** n=6**)** in the same co-culture systems **(l and m,** n=6**)**. While also decreasing B-cell mediated candida specific T cell expression of pro-inflammatory (IFNγ and IL-17) cytokines (**n-r**, n=6). *$p<0.05$, **$p<0.01$ and ***$p<0.001$.

Supplementary Fig. 6

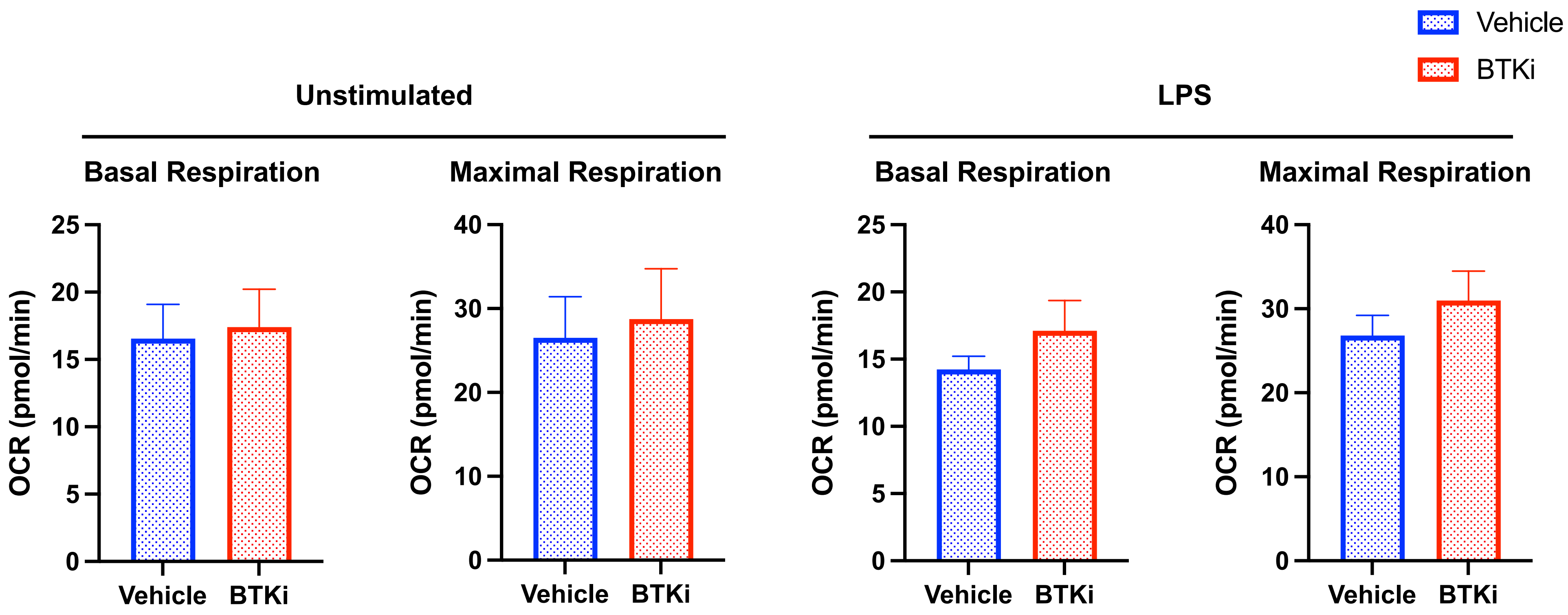

Supplementary Fig. 6. Unlike its effect on B cells, BTKi does not decrease mitochondrial respiration in monocyte-derived macrophage.

Human $CD14^+$ monocyte-derived macrophage were generated using M-CSF over 6 days in culture, then kept either Unstimulated or stimulated with LPS, with the addition of Vehicle or BTKi 30 min before the addition of LPS. After 12hrs, Mitochondrial respiration was measured using seahorse assay. BTKi does not alter mitochondrial respiration of macrophage under basal culture conditions or upon LPS stimulation.

Supplementary Fig. 7

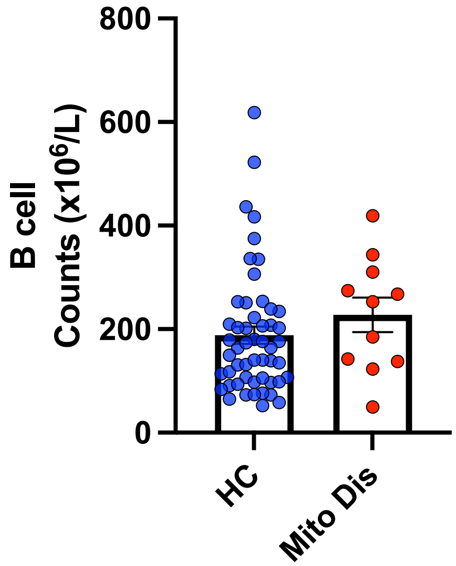

Supplementary Fig. 7. Mitochondrial deficiency does not impact circulating B cell counts.

Flow cytometry was used to measure circulating B cell counts in blood samples obtained from patients with known mitochondrial complex I (ND3 or ND6) or V (USMG5) respiratory chain mutations (n=11; Table. S1) or healthy controls (n=48). B cells counts did not appear to be affected by the mitochondrial mutations.

Supplementary Fig. 8

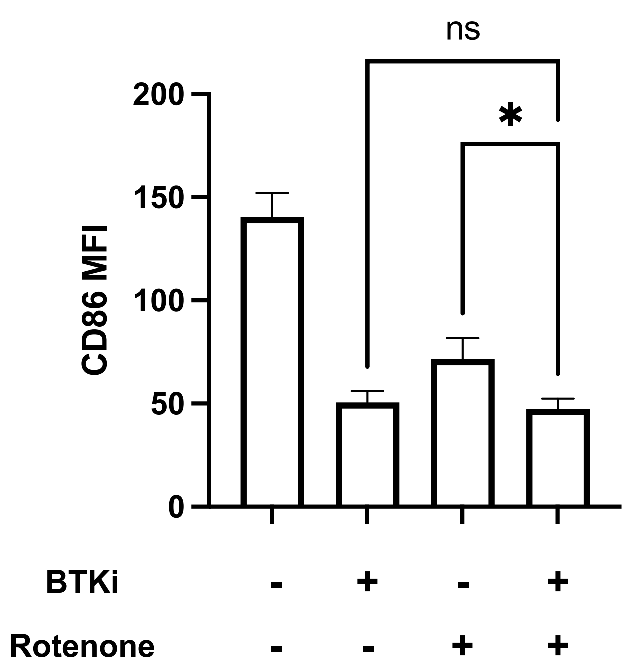

Supplementary Fig. 8. No additive inhibitory effect when combining inhibition of mitochondrial respiration with BTKi.

Human B cells were treated with either BTKi or rotenone alone, or in combination, for 30min and then stimulated with BCR cross-linking antibodies for 36hrs. CD86 were measured by flow cytometry (n=7). Blocking mitochondrial respiration did not further decrease costimulatory molecule expression in presence of BTKi. These data suggest that BTKi modulation of B cell co-stimulatory molecule expression mat be mediated at least in part through mitochondrial respiration. Repeated measure Two-way ANOVA; **p<0.01 ***p<0.001 and ****p<0.0001

Supplementary Fig. 9

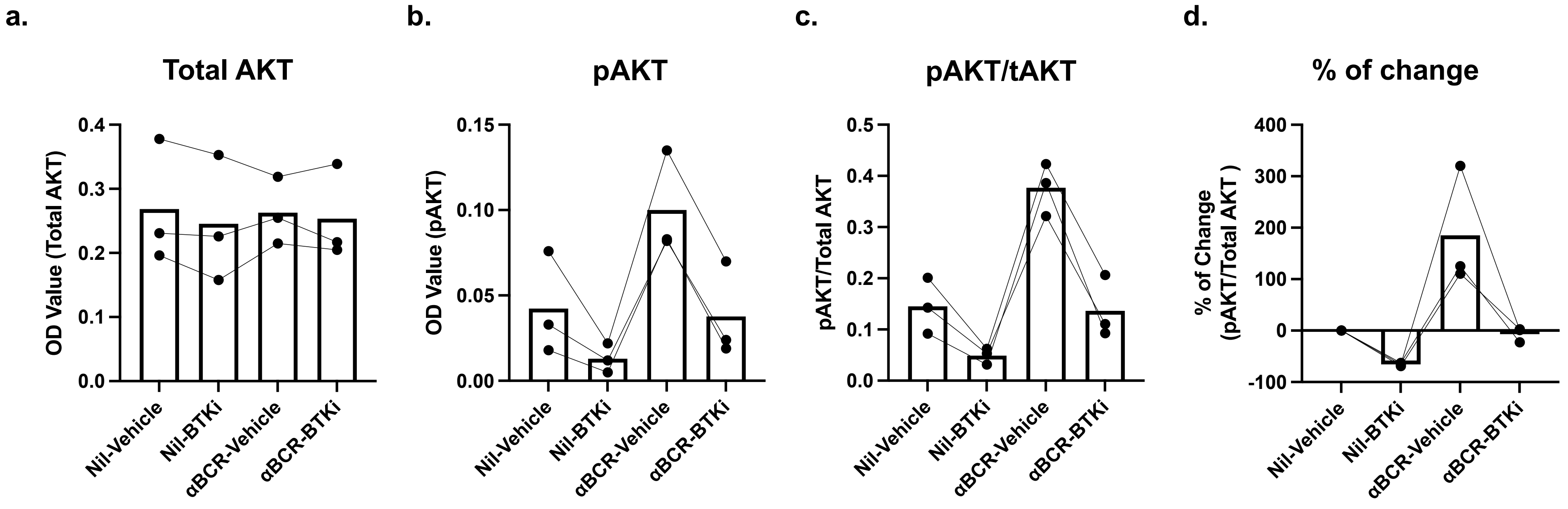

Supplementary Fig. 9. BTKi decreases phosphorylation of AKT.

Human B cells were treated with BTKi for 30min and then stimulated with BCR cross-linking antibodies for 15min. Total AKT (tAKT) and phosphorylated AKT (pAKT) were measured by ELISA. BTKi decreases pAKT without affecting the expression of total AKT. (n=3)

Supplementary Fig. 10

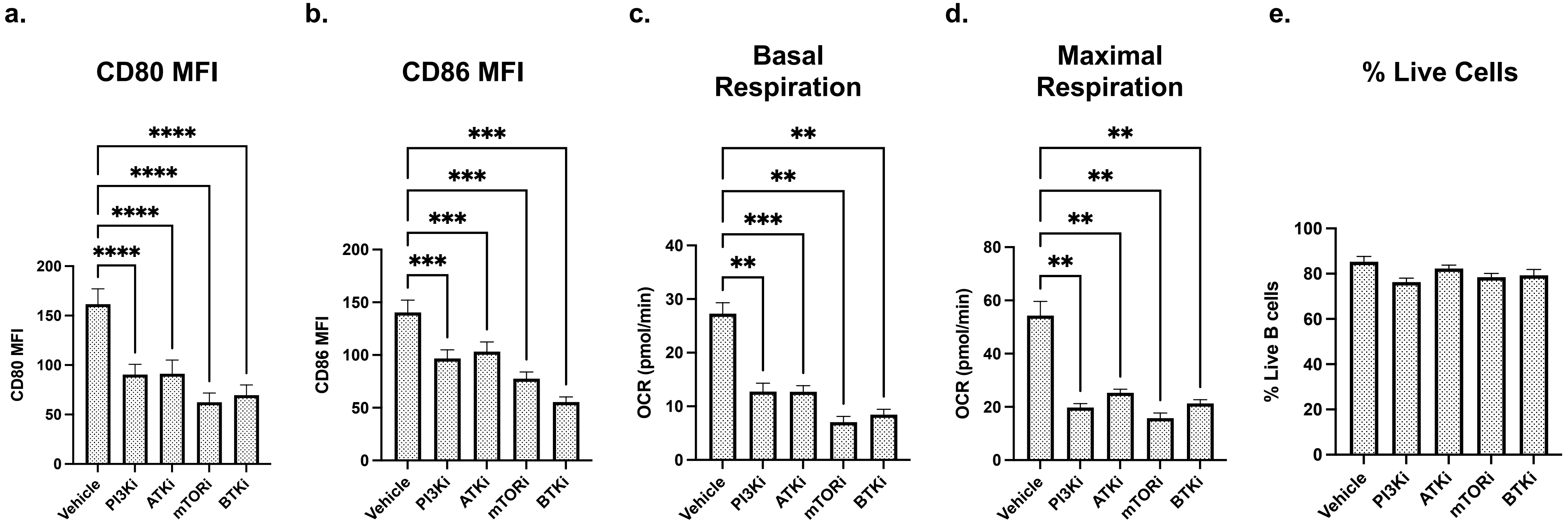

Supplementary Fig. 10. The blockade of multiple elements of the PI3K-AKT-mTOR pathway modulates B-cell costimulatory molecule expression as well as mitochondrial respiration.

Human B cells were treated with various inhibitors of the PI3K-AKT-mTOR pathway or BTKi for 30min and then stimulated with BCR cross-linking antibodies for 36hrs. Expression of CD80 (a) and CD86 (b) was measured by flow cytometry. Mitochondrial basal (c) and maximal (d) respiration were measured by seahorse mitostress assay. Inhibition of the PI3K-AKT-mTOR pathway reduces B cell costimulatory molecule expression, which is associated with a decrease of mitochondrial respiration. (n=7) Repeated measure One-way ANOVA; **p<0.01 ***p<0.001 and ****p<0.0001.

Supplementary Fig. 11

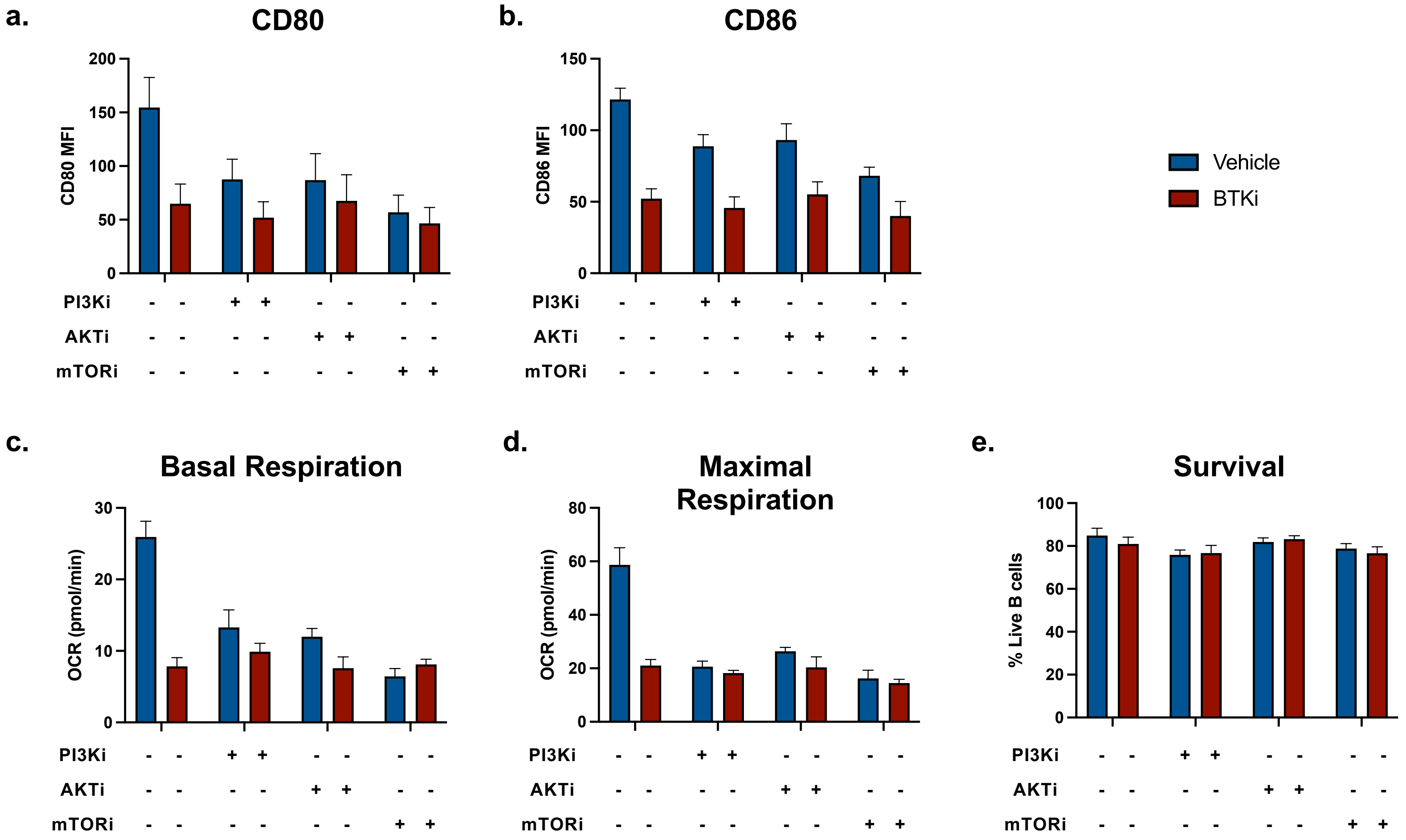

Supplementary Fig. 11. No additive inhibitory effect when adding inhibition of either PI3K, AKT or mTOR to inhibition of BTK. Human B cells were treated with various inhibitors of the PI3K-AKT-mTOR pathway in addition to BTKi for 30min and then stimulated with BCR cross-linking antibodies for 36hrs. (a-e, n=4). Blocking elements of the PI3K-AKT-mTOR pathway did not further decrease costimulatory molecule expression or OXPHOS in presence of BTKi, suggesting that BTKi may regulate B-cell metabolism and co-stimulatory molecule expression through (or at least partially through) the PI3K/AKT/mTOR pathway. modulation of B cell co-stimulatory molecule expression is mediated partially through PI3K-AKT-mTOR pathway.

Table S1. Demographic Table of patients with mitochondrial complex deficiencies

Patient ID	Age	Gender	Mutation	Diagnosis
CHOP-1	14	Female	ND3 Mutation	MELAS
CHOP-2	19	Male	ND3 Mutation	MELAS
CHOP-3	23	Male	ND3 Mutation	KSS
CHOP-4	13	Female	ND3 Mutation	LHOH
CHOP-5	17	Female	ND3 Mutation	MELAS
CHOP-6	14	Male	ND6 Mutation	KSS
CHOP-7	15	Male	ND3 Mutation	KSS
CHOP-8	13	Male	ND3 Mutation	MELAS
CHOP-9	15	Male	USMG5 Mutation	MELAS
CHOP-10	67	Female	ND3 Mutation	MELAS
CHOP-11	45	Male	ND3 Mutation	MELAS

Table S2. Demographic Table for MS Patients and Healthy Control Participants

	HC	RRMS
Number	14	16
Age (Mean ± SD)	38.6 ± 13.6	38.9 ± 8.37
Gender (F/M)	11:3	12:4
Average Disease duration from Diagnosis	N/A	8 weeks
Treatment status	N/A	Never Treated
